# To Die or Not to Die—Regulated Cell Death and Survival in Cyanobacteria

**DOI:** 10.3390/microorganisms10081657

**Published:** 2022-08-17

**Authors:** Natasha S. Barteneva, Ayagoz Meirkhanova, Dmitry Malashenkov, Ivan A. Vorobjev

**Affiliations:** Department of Biology, School of Sciences and Humanities, Nazarbayev University, Nur-Sultan 000010, Kazakhstan

**Keywords:** cyanobacteria, regulated cell death, regulated cell survival, multicellularity, single cell analysis, cyanophages, image-based cell sorting, environmental stress

## Abstract

Regulated cell death (RCD) is central to the development, integrity, and functionality of multicellular organisms. In the last decade, evidence has accumulated that RCD is a universal phenomenon in all life domains. Cyanobacteria are of specific interest due to their importance in aquatic and terrestrial habitats and their role as primary producers in global nutrient cycling. Current knowledge on cyanobacterial RCD is based mainly on biochemical and morphological observations, often by methods directly transferred from vertebrate research and with limited understanding of the molecular genetic basis. However, the metabolism of different cyanobacteria groups relies on photosynthesis and nitrogen fixation, whereas mitochondria are the central executioner of cell death in vertebrates. Moreover, cyanobacteria chosen as biological models in RCD studies are mainly colonial or filamentous multicellular organisms. On the other hand, unicellular cyanobacteria have regulated programs of cellular survival (RCS) such as chlorosis and post-chlorosis resuscitation. The co-existence of different genetically regulated programs in cyanobacterial populations may have been a top engine in life diversification. Development of cyanobacteria-specific methods for identification and characterization of RCD and wider use of single-cell analysis combined with intelligent image-based cell sorting and metagenomics would shed more light on the underlying molecular mechanisms and help us to address the complex colonial interactions during these events. In this review, we focus on the functional implications of RCD in cyanobacterial communities.

## 1. Introduction

Genetically regulated cell death (RCD) was first identified in metazoans and later found in prokaryotes and different eukaryotes [1,2,3]. The multiple forms of RCD execution seem to involve a series of stereotypical morphological and biochemical hallmarks such as initiation of proteolytic cascade and DNA degradation. Apoptosis-like RCD in metazoans is characterized by lipid rearrangement of the plasma membrane and a decrease in cellular volume and changes in ultrastructure, genome fragmentation, and at the final stages, disassembly of the cell into membrane-enclosed vesicles. Research on RCD has made spectacular progress in eukaryotes, including unicellular organisms [4,5,6,7,8]. In the last decade, emerging evidence suggests that RCD-linked genes and activities are also possibly involved in bacterial life cycle regulation and environmental stress adaptations [1,9,10,11], stress-related protein complexes in archaea [12,13,14], and cyanobacteria [15,16,17]. Many hypotheses have been proposed as to why RCD evolved in unicellular prokaryotes despite its obvious disadvantage to individual fitness. The possible evolutionary advantages (i.e., at the population level) can be related to (a) better response to stress and starvation, (b) immunity against pathogens, and (c) development of transiently multicellular organisms [3]. RCD in prokaryotes is triggered by environmental stresses (nutrient deprivation, oxidative stress, high salinity, high light levels, heavy metals pollution) and biotic factors (infection with pathogens, allelopathy) and may lead to autocatalytic cell suicide and, eventually, cell demolition. It is related to massive cyanobacterial cell death and lysis in nature [18]. Importantly, (1) cell death in cyanobacteria is regulated and is not just an accidental event because of harsh environmental conditions or stress and (2) in addition to morphological criteria for RCD identification and characterization, there is increasing molecular evidence about the RCD-like sequence of events in cyanobacteria. 

However, a stress response in different cyanobacteria species also relies on regulated cellular survival (RCS) programs such as chlorosis and resuscitation, leading to temporary dormancy in the unicellular non-diazotrophic cyanobacteria [19] and to the maturation of akinetes in heterocyst-forming multicellular filamentous cyanobacteria [20]. Cyanobacteria are one of the most morphologically diverse prokaryotic phyla where multicellularity has evolved [21]. Cyanobacteria represent a group of oxygenic phototrophic bacteria with Gram-negative characteristics, several members of which are colonial (*Microcystis*) and/or exhibit multicellularity, with up to six distinct cell types in filamentous cyanobacteria [22,23]. Taxonomic research traditionally organizes the cyanobacteria into five subsections based on their morphological traits [24]. Unicellular forms belong to subsection I (Chroococcales), which undergo binary fission, and subsection II (Pleurococcales), which reproduce through multiple fissions in three planes. Evolutionary sequencing-based studies suggested that within the cyanobacterial phylum, reversions and switches from filamentous multi- to unicellularity occurred, affecting a marine planktonic *SynPro* clade and *Gloeocapsopsis* clade that has been traditionally classified in subsection II [25,26]. The transition to multicellularity is a major evolutionary transition, and multicellular life forms have evolved independently many times, suggesting multiple advantages associated with this transition [27]. Nitrogen fixation was found to be the prime driver of multicellularity in cyanobacteria [23], and a trade-off between nitrogen fixation and photosynthesis led to the compartmentalization of these two functions through multiple evolutionary solutions [23].

**Figure 1:**
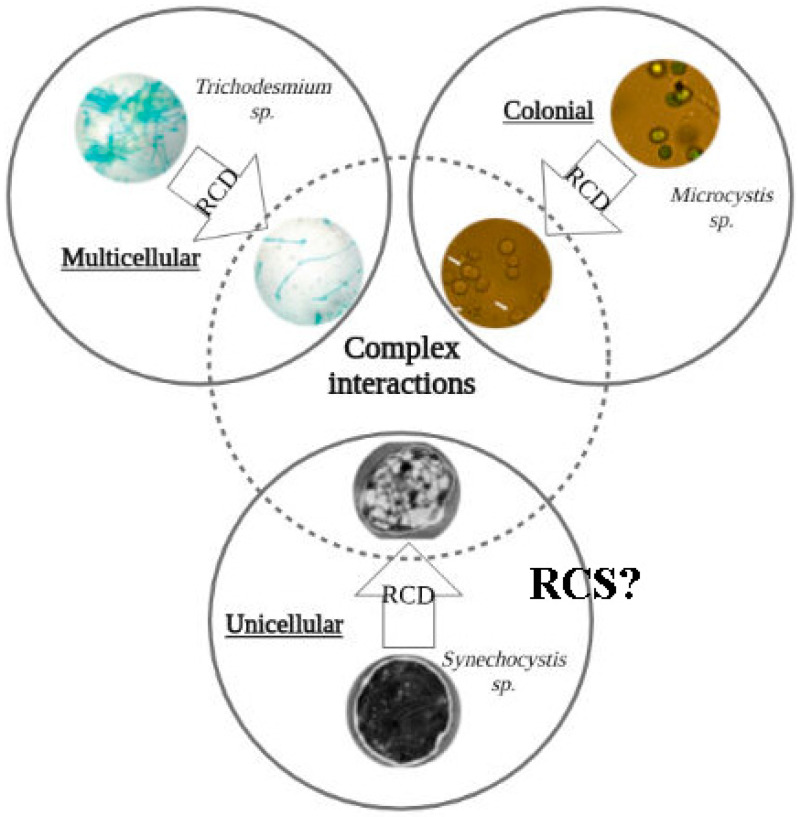
Regulated cell death (RCD) models in cyanobacteria. Regulated cell death (RCD) has extensively been studied in a few biological models. It includes multicellular (filamentous) cyanobacteria, such as *Trichodesmium* spp. (**top left**). RCD was also documented in colonial strains and in some unicellular organisms, which was recently suggested could reverse multicellularity (*Microcystis* spp.—**top right**, *Synechocystis* spp.—**bottom**).

RCD in cyanobacteria is a long-studied phenomenon mainly in multicellular cyanobacteria, in colonial organisms (*Microcystis* sp.), and in cyanobacteria species that were considered to be unicellular forms but probably at a certain evolutionary point reversed from multicellularity to unicellularity ([28,29,30,31,32,33,34,35,36,37,38,39,40,41,42,43,44,45,46,47,48,49,50,51,52,53,54,55,56,57,58,59,60,61,62,63,64], Table 1, Figure 1). Some representatives of section I of cyanobacteria form enclosed sheath cellular aggregates, and some section II representatives form multicellular aggregates enclosed by an additional fibrous layer [23,65].

All this brings us to a question: if practically all studies of RCD in cyanobacteria were performed on multicellular and colonial (at least temporary multicellular) species—what happens with unicellular cyanobacteria in response to the environmental stress? Does their stress response initiate the regulated programs of survival (RCS) following resuscitation or regulated cell death? Opposite to multicellular cyanobacteria RCD research, practically all studies on chlorosis and RCS were conducted on two non-diazotrophic unicellular model species (*Synechocystis* spp. and *Synechococcus elongatus*) [19]. For unicellular non-diazotrophic cyanobacteria, exit to reversible dormancy and quiescence by a regulated survival program may be a key to rescue, though co-existence of cells executing RCD and RCS and heterogeneity of cyanobacterial populations along the quiescence–RCD response will provide an advantage at the population level.

Limitations of existing methods to study RCD in cyanobacteria are currently actively discussed (ref. [66]). Methods for studying RCD were developed to investigate vertebrate cells where metabolic changes during apoptosis rely on mitochondria as stress sensors and central executioners of cell death [67,68,69,70]. Therefore, the direct transfer of existing vertebrate methodology to cyanobacteria organisms that have no mitochondria and are using different sources of energy—photosynthesis and nitrogen fixation—may lead to the false findings introduced by analogical reasoning [71].

In this study, we review functional applications of RCD in cyanobacteria in relation to abiotic and biotic (cyanophages and other pathogens) factors.

## 2. Functional Importance of RCD in Cyanobacteria

There is direct experimental evidence for RCD-associated benefits for higher-group-level prokaryotes, such as survival under phage infection. During infection with a pathogen, a prokaryotic strain capable of RCD outperforms non-RCD strain, indicating surplus at the population level [72]. One of the first routes to multicellularity was to aggregate temporarily in order to resist unfavorable conditions [73]. The colonial cyanobacteria such as *Microcystis* spp., at least transiently, are part of multicellular systems, and some cyanobacteria, such as diazotrophic filamentous spp. are functionally differentiated (akinetes, heterocytes, vegetative cells) [74]. A bacterial community can induce RCD in a part of population to favor the colony survival in response to the external stress stimulus: phage and fungal infections, nutrient deprivation, radiation exposure and oxidative stress [75]. However, the cell specialization and RCD co-evolved together with other pathways of survival—the ability to develop metabolic rewiring and reorganization of cellular machinery leading to cell dormancy and reversible quiescence [76]. Remodeling of photosynthetic apparatus and cellular metabolic machinery reorganization accompany chlorosis and cellular survival and resuscitation after long-term chlorosis [77].

### 2.1. Starvation

One of the explanations for bacterial RCD appearance during evolution is a mechanism for functional interaction between unicellular organisms, which further increases their fitness via mutual dependence [78]. Under stress conditions, as a result of nutrient starvation, dying cyanobacteria release nutrients essential for the survival of the remaining population (“nutrient acquisition”) [79,80,81]. In 2004, a study reported an autocatalytic RCD in *Trichodesmium* spp. in response to nutrient starvation [16]. It happens, for example, in colonial morphospecies when nutrients for the entire colony are limited, and the cell death of some cells may increase the chances for colony survival—similar to results obtained with colonial yeasts [82,83]. Bar-Zeev and colleagues [39] demonstrated the release of transparent polysaccharides during RCD in *Trichodesmium* algal blooms and the consequent downward flux of nutrients, supplying the bloom participants with carbon and nitrogen. RCD, in this case, facilitates the nutrient cycling in the microbial loop—how a unicellular organism dies was shown to affect the fate of other microorganisms [2]. In particular, the exchange of nutrients released upon the lysis of a photosynthetic organism led to the recycling of these dissolved organic materials (DOMs). 

Cyanobacteria evolved under periodic changes between periods of nutrient sufficiency, allowing fast growth, and the following periods of nitrogen depletion when the cells needed to cope with starvation until nutrients were replenished. However, the story of nitrogen starvation shaping cyanobacteria functionality and evolution is rather complicated, and we will briefly discuss just a few aspects of this complex story related to regulated cell survival (RCS) (we recommend an excellent specific review for detailed discussion on the mechanisms of nitrogen starvation and chlorosis [19]). Two major metabolic processes in cyanobacteria are the processes of photosynthesis and N_2_ fixation. They are regulated to increase cellular fitness and ecological competitiveness by distinct daily cycles such as circadian rhythms [84,85]. Oscillation changes in photosynthesis and nitrogen fixation were found initially in several diazotrophic cyanobacteria strains [86,87], and later Huang and co-authors [88] discovered the classical circadian rhythm of nitrogen fixation in *Synechococcus* sp. RF-1. Cyanobacteria are the only prokaryotes known to possess a circadian clock [84]. 

The circadian clock in *Synechococcus elongatus* comprises the core oscillator proteins KaiA, B, and C [84]. Under light–dark (LD) conditions *S. elongatus* employs photosynthesis and carbon fixation, and stores fixed carbon as glycogen [89]. In the dark period, glycogen is rapidly degraded via the oxidative pentose phosphate pathway. Regulator of phycobilisome (PBS) association (RpaA) was first identified as a protein that influences the ratio of light energy transfer from PBS to photosystems PSI vs. PSII [90]. RpaA forms a two-component pair with SasA histidine kinase, which interacts directly with central circadian core components (KaiC). The RpaB forms a two-component pair with the histidine kinase Hik33 and participates in the responses to environmental stress such as oxidative stress, high salinity, and high light [91]. Most of the insight into the functionality of this two-component system, as we already mentioned, was obtained using only two model cyanobacteria species—*Synechocystis* 6803 and *Synechococcus elongatus PCC 7942.* Typical cyanobacteria possess multiple two-component systems, mainly comprising a membrane-bound histidine kinase (HK) and its cognate cytoplasmic response regulator. Their quantity is widely varied—from just 11 putative genes encoding two-component systems in the genome of *Prochlorococcus* MED4, up to 146 HK and 168 genome regulators (RR) in filamentous *Nostoc punctiforme* [92,93]. Recently, the possibility of direct thiol regulation of some cyanobacterial RRs (Rre1, RpaA, and RpaB) by the redox state of the photosynthetic electron transport chain through ferredoxin or thioredoxin was suggested [94]. Moreover, the mutants of *S. elongatus* defective for the *rpaA* accumulate excessive reactive oxygen species (ROS) during the day and are inviable after several hours in the dark, likely because NADPH is required to detoxify ROS, and enzymes of the NADPH pathway are direct targets of RpaA [95].

The need to modulate the photosynthetic apparatus between alternative metabolic conditions is responsible for developing of the genetically regulated process of chlorosis associated with the response to nitrogenstarvation (rev. [19]). Chlorosis is characterized by the degradation of photosynthetic pigments and is accompanied by a color change from blue green to yellow (hence the name) [96]. The chlorosis (bleaching of algal cultures in response to nitrogen starvation) was already described in 1910 [97]. Phycobilisomes may account for up to 60% of the cellular protein in the cyanobacterial cell and serve as a major cellular reserve in nutrient starvation conditions [98]. Upon nitrogen depletion, phycobilisomes—giant protein–pigment complexes anchored to the thylakoid membranes—rapidly degrade following a regulated genetic program [99,100,101]. Degradation of PBS supplies nutrients for cellular metabolism during nutrient deprivation conditions, prevents photosynthetic apparatus from undergoing photoinhibition and production of harmful ROS [102], and plays an important role in cell survival [103]. Following long-term nitrogen deprivation, chlorophyll (Chl) level decreases dramatically to <1% of the original [104]. Using *Synechocystis* PCC 6803 sp. as a model, Murton and co-authors [103] demonstrated heterogeneity of pigment distribution in the single cells that can be explained by the self-shading effect [105] and revealed clustering of cells with high PC and low Chl–PSII in the concerted population response to nitrogen starvation. A first comprehensive analysis of a genetically regulated program for resuscitation from chlorosis was published by Klotz and co-authors [106] using *Synechocystis* as a model. When long-term chlorotic *Synechocystis* cells are supplied with nitrogen, they regain photosynthetic activity after about 24 h, and cell division resumes after three days. The transcriptomic and proteomic analysis revealed that during this lag-phase period, cyanobacterial cells are rebuilding the complex photosynthetic machinery [106,107]. In the different resuscitation phases, cyanobacterial cells go through different metabolic phases—from heterotrophic-like metabolism based on glycogen consumption and oxygen-dependent respiration to a mixotrophic metabolism when glycogen assimilation co-occurs with oxygenic photosynthesis, and finally—to the resumption of classical photoautotrophic metabolism [77]. Photosynthetic apparatus remodeling and cellular metabolic machinery reorganization accompany chlorosis and resuscitation after long-term chlorosis. Importantly, it is a genetically regulated program of cellular survival (regulated cellular survival or RCS). During the first steps of the resuscitation of unicellular cyanobacteria, an almost instantaneous (in minutes) increase in the ATP level is observed [77]. 

### 2.2. Differentiation and RCD in Cyanobacteria

Complex multicellularity evolved app. 2.5 billion years ago, and since then has been lost and regained several times in cyanobacteria [18,108,109]. In particular, filamentous cyanobacteria may undergo cellular differentiation into at least five specialized cell types: photosynthetic vegetative (photosynthetic) cells, nitrogen-fixating heterocytes (=heterocysts), spore-like cells—akinetes [74hormogonia cells, and necridia. Moreover, vegetative cells can divide and generate other cell types, but heterocytes cannot revert back and thus represent terminally differentiated cells [110]. Apart from nutritional function, heterocytes also regulate the placement and positioning of akinetes [111]. Hormogonia represent another cyanobacterial cell type characterized as short, motile filaments [74], the primary function of which is the colonization of new environments more suitable for growth [112]. The release of hormogonium from the parental trichome happens after the formation of released dead cells, called necridia [112,113,114,115].

Necridia transform and adapt themselves for their final function, the attachment of hormogonia to the colonized surface. This mechanism can act in conjunction with other adhesion mechanisms. The first stage in necridia differentiation is characterized by a peak in fluorescence that accompanies the loss of photosystem II activity, which is well described in heterocytes [116]. The appearance of these highly fluorescent cells allows the fragmented filaments to escape unfavorable environmental conditions. Depending on the mechanical strain, cell bleaching is followed by the breaking of the parental filament. The nonrandom position of the high-fluorescence cell within the filament, the abrupt appearance of the elevated chlorophyll a fluorescence, persisting photochemical activity, and the equally abrupt decomposition of the photosynthetic apparatus indicate that cell death is strongly controlled rather than being an accidental decay process induced by unfavorable environmental conditions [112]. The alternative strategy was developed by the cyanobacteria *Plectonema boryanum* (=*Leptolyngbya boryana*), where it switches back and forth between nitrogen and carbon fixation, hence separating processes not in space but in time, i.e., achieving temporal differentiation [117]. 

Cell death of filamentous cyanobacteria of different genera in exposure to stress leads to fragmentation of filaments and the subsequent autolysis of cells. The extrusion of cellular components and loss of viability that is observed in *Anabaena variabilis* during heterocyst differentiation can also be considered as evidence for the role of RCD in cell propagation and dispersal [78]. Environmental stress triggers akinete formation and induces reactive oxygen species (ROS) accumulation in the cyanobacterium *Aphanizomenon ovalisporum (=Chrysosporum ovalisporum)* [118]. However, the differentiating *Aphanizomenon* cells prevent the oxidative burst by degradation of the antioxidative machinery and phycobilisome antenna [118,119]. 

Differentiation of vegetative cells to dormant forms (akinetes) in cyanobacteria (Nostocales and Stigonematales orders) has been studied since 1856 [120]. Significant progress in analyzing mechanisms that lead to the akinete formation has been achieved recently [76,77]. Only heterocyst-forming filamentous multicellular cyanobacteria form akinetes, and their differentiation and maturation have been studied in detail using two model organisms: the terrestrial *Nostoc punctiforme* ATCC 29133 and the freshwater *Anabaena variabilis* (=*Trichormus variabilis*) ATCC 29413. The environmental stresses, such as nutrient starvation, temperature changes, and light availability, trigger akinete germination [20,121]. This process begins with a reorganization of the cellular material, followed by elongation and cell division that occur inside the akinete envelope [122]. Despite the last decade's developments, precise molecular and cellular mechanisms involved in the germination of akinetes remain unclear.

### 2.3. Infection-Triggered Response in Cyanobacteria Is Activated Coordinately with Multiple Defense Functions

Cyanobacteria co-exist under natural conditions with other organisms such as cyanophages, heterotrophic bacteria, protists, and algicidal microfungi [123]. Besides grazers, the parasites participate in top-down control of phytoplankton [124,125] with the coexistence of several parasites with the same target host. Thus, Manage and co-authors [126] demonstrated the coexistence of the algicidal bacteria *Alcaligenes denitrificans* and cyanophages-like particles, which simultaneously participated in the regulation of *Microcystis aeruginosa* blooms. Cyanobacteria colonial forms may have even more associated organisms, including bacteria, microalgae (diatoms, chrysophytes, and dinoflagellates), and metazoans (hydroids, juvenile copepods, and decapods) [127]. The colonial structural organization and dissolved nitrogen and carbon organic substances provided by colonies encourage the association with heterotrophic organisms. 

Could RCD evolve in colonial and unicellular cyanobacteria as an antipathogen strategy? Given a high abundance of viruses in diverse ecosystems [128,129,130,131] and a co-evolution of viruses with cellular life forms since the earliest stages of life, some forms of RCD might have evolved from antivirus defense machinery.

#### 2.3.1. Cyanophages and RCD in Cyanobacteria

Thus, RCD is thought to be a defense mechanism upon the initiation of which cyanobacterial cells can resist phage infection [132]. Viruses are the most abundant biological entities in the ocean, and through horizontal gene transfer and lysis of specific hosts, cyanophages influence bacterial community diversity and composition [133,134]. The genetic diversity of viruses may substantially exceed the diversity of cellular-like organisms [135]. Natural blooms often terminate abruptly, and cyanophages may affect bloom demise significantly [16]. By lysing cyanobacteria, cyanophages may cause the collapse of cyanobacterial blooms and potentially serve as biological agents to control the cyanobacteria-induced HABs [136]. 

The filamentous freshwater cyanobacteria and well-studied marine cyanobacteria significantly differ in the size of their genomes (from 3.88 Mb *Cylindrospermopsis* spp. to 9.06 Mb *Nostoc* spp. for freshwater vs. 1.6–2.7 Mb for some sequenced *Prochlorococcus* genomes [137] and 2.1–3.7 Mb for sequenced marine *Synechococcus* spp. genomes [138]) and cellular size. Chytrids prefer large hosts to small ones [139,140,141,142]. The difference in the cell sizes in freshwater and marine counterparts make cyanophages, and perhaps, heterotrophic bacteria but not fungi (chytrids), major regulators of ocean cyanobacterial blooms. 

The current understanding of viral metagenomics and culturing experiments in model systems is that phages hijack host cell metabolism and increase host fitness during frequent periods of nutrient limitation [143]. The virally encoded host-like proteins could have a role in restoring protein synthesis in a starved cell to allow for virus replication and delay RCD. So far, RCD is thought to be an antiviral defense mechanism, upon the initiation of which cyanobacterial cells can withstand phage infection [132]. 

**Figure 2:**
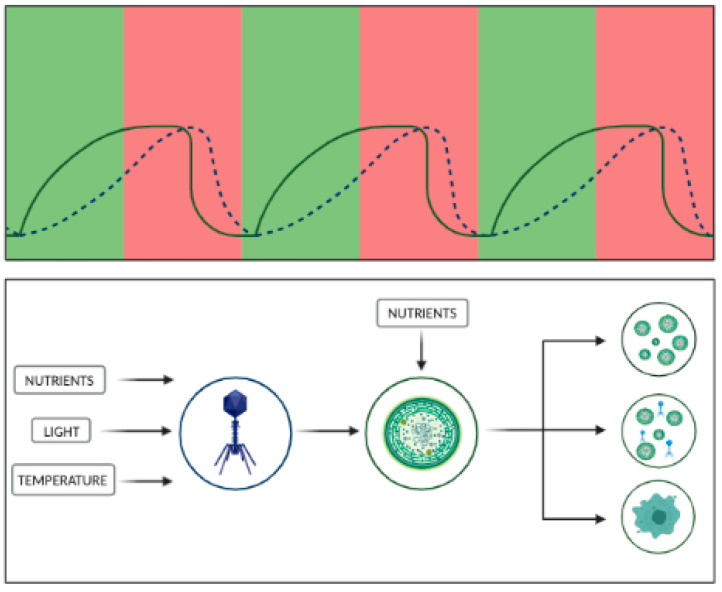
Cyanophages and nutrient cycling in cyanobacteria. The viral infection of algae can result in multiple cell fates. According to the diagram, part of the population would be metabolically active while still being infected, serving as viral particle releasing machines; the second part of the population would be lysed upon the infection, releasing organic matter; lastly, a part of the population would be resistant to the infection, thus serving as an inoculum for future populations. On top of this, the cycle of cyanobacterial population growth is also closely linked to the level of available nutrients. This relationship (similar to a typical predator–prey curve) can thus be described as follows, high-nutrient phases are characterized by progressive growth of algal populations (solid line), which in turn leads to the progressive growth of viruses (dotted line). Upon reaching a certain growth point, the cyanobacterial population experiences nutrient limitation, as late phases of algal blooms have been demonstrated to have low levels of available P and N. Under such conditions, phages can utilize cellular host machinery in order to maintain the phage population, leading to (1) release of viral particles for maintenance of the phage population, (2) release of organic matter for further decomposition by microbes, and (3) development of a resistant cyanobacterial population for the beginning of a new cycle.

Distinct transcriptional states may co-exist during the active viral infection of cyanobacteria: (1) cells resistant to lysis that keep persisting viruses (“persisters”) and could potentially serve as an inoculum for next blooms; (2) metabolically active infected cells responsible for viral release followed by lysis, and (3) a population of cells dying without releasing any viral particles (Figure 2). Similar observations were made by Tucker and Pollard [144] on how a *Microcystis aeruginosa* population recovered from a cyanophage infection in about three weeks, possibly due to a developed resistance. So, it is reasonable to assume that cyanophage infection could ultimately result in diverse cell fates in the bacterial host. Because late-phase blooms are characterized by low [P] and [N] levels, phage infection could be activated, and cyanophage would actively utilize host cellular machinery to maintain and expand the phage population [16]. At the same time, the release of organic material during lysis would contribute to the decomposition of organic matter by heterotrophic microbes [145]. Fuchsman and co-authors [136] have proposed a similar mechanism for *Prochlorococcus*, and Bar-Zeev et al. [39] for a nitrogen-fixing cyanobacterium, *Trichodesmium*. In the latter study, recently fixed nitrogen was sinking into the sediments with the cell for further decomposition by heterotrophic bacteria. 

However, it is unclear what the primary factors that stabilize the host–phage relationship are and whether this relationship is cyclic [146]. Light and photosynthetic processes strongly influence the success of phage infection and the number of generated cyanophage particles and their release [147]. Multiple studies have identified photosynthetic “host” genes present in the phage genome, which are hypothesized to be significant during phage infections as they increase the physiological and ecological fitness of infected cyanobacteria [148]. One of these examples, the *speD* gene—a homolog found in all marine cyanobacteria—is responsible for polyamine biosynthesis. Along with the *psbA* gene, also present in the marine phage genome [149], if expressed, *speD* is thought to maintain the activity of the host photosystem II reaction center during phage infection [133,148]. It is hypothesized that phages perturb cyanobacteria metabolism to keep them alive for a more extended period stimulating phage multiplication.

#### 2.3.2. Bacteria

Bloom-forming cyanobacteria are closely associated with heterotrophic bacteria [150,151,152,153,154]. Multiple sampling across continents confirms the phylogenetic and functional similarity of *Microcystis*-associated bacteria and the existence of complementary pathways between *Microcystis* and associated heterotrophs [155]. The heterotrophic bacterial community changes when it participates in cyanobacteria blooms. Thus, Shi and co-authors [156] observed that *Rhodospirillales*, *Burkholderiales*, and *Verrucomicrobiales* dominated during the rapid decomposition phase, whereas *Sphingomonadales*, *Rhizobiales*, and *Xanthomonadales* dominated during the slow decomposition phase. Changes in a bacterial community are likely involved in the formation and maintenance of *Microcystis* colonial morphoforms [157]. These findings are in accord with reports that heterotrophic bacteria secrete extracellular components that affect the metabolism and growth of cyanobacteria [152,158,159]. In nature, and even when recently isolated *Microcystis* was maintained in the lab culture, colonies contain heterotrophic bacteria [160]. Furthermore, bacteria of the genus *Sphingomonas* are associated with *Microcystis* blooms and actively break down toxins [161,162]. Several bacterial strains were found to inhibit or enhance the growth of associated cyanobacteria [163]. Recent studies by Jankowiak and Gobler [164] confirmed that *Microcystis* colonies harbor bacterial assemblages that may be conserved across geographically distinct blooms and over different stages of blooms [164]. This finding also suggests that *Microcystis* colonies harbor a selected pool of bacteria with a conserved functional potential that may help bloom persistence even under unfavorable environmental conditions. Parasite strategies, such as bacterial regulation of host RCD, can drive the evolution of host–parasite interactions leading to a possible outcome of cell death, dormancy, or infection. If bacteria delay and/or promote *Microcystis* colony growth and participate in colony decay, they may participate more actively in inducing cell death in the old colonies.

#### 2.3.3. Fungi

In addition to well-studied viruses, many cyanobacteria species are affected by fungal parasites mainly belonging to Chytridiomycota (chytrids) [165]. These microfungi infect their host cells by zoospores that digest the cytoplasm, eventually form sporangia, and finally produce and release new zoospores [125]. Chytrid parasites of cyanobacteria are ubiquitous, undergo strong variations from year to year, and may burst into epidemics, reaching a prevalence of infection up to 90–98% [142,166]. Chytrids infect different freshwater genera of cyanobacteria, including *Anabaena*, *Aphanizomenon*, *Cylindrospermopsis*, other filamentous taxa, and *Microcystis* [123,125,167]. Chytrids infecting cyanobacteria are obligate parasites, and many of them have narrow host limits and cannot use alternative food sources [168]. In diatoms (*Asterionella formosa*), RCD accelerates the death of infected host cells and the chytrid parasite before it completes its life cycle [169]. A fungal infection may also reduce the length of cyanobacterial filaments by so-called “mechanistic fragmentation” [170] and, therefore, may promote bloom decline by enhancing grazing [171] and facilitating host–parasite dispersal [172]. However, it is unclear if the “mechanistic fragmentation” of cyanobacterial filaments happens because of the RCD-like mechanism participating in releasing uninfected parts of the filament. 

## 3. Methods to Study RCD in Cyanobacteria

Early examination of phytoplankton automortality characterized by a loss of membrane integrity and degradation of photosynthetic activity, led scientists to supposition of a genetically based process of RCD as a source of phytoplankton mortality [15]. Current research on cyanobacterial RCD [50,69,72,73] concentrates on several morphological and biochemical features parallel to those found in eukaryotic RCD, including ultrastructure changes and cell membrane rearrangement, and activation of proteolytic enzymes [132,173]. 

Initially, the hallmarks of RCD in cyanobacteria were meant to be visualized using a standard for mammalian apoptosis reagents and techniques, such as labeling cell membrane with fluorochrome-conjugated annexin V to verify exposure of phosphatidylserine (PS) ([43,72,173], Table 1). During apoptosis in eukaryotes, the annexin V signal parallels effector caspase activation [68]. Positive staining with annexin V was demonstrated in cyanobacterial akinetes, heterocytes, and vegetative cells. However, peptidoglycan, a key component of a prokaryotic cell wall, can be an obstruction in the staining of phosphatidylcholine with annexin V [174]. Also, the shrinking/changing volume of the cell, which is considered an early sign of RCD in the mammalian cell, is limited due to a rigid peptidoglycan wall. In cyanobacteria, the peptidoglycan’s layer status may serve as an important indicator of living/dead status [72]. Cyanobacterial viability was estimated by the ratio of dead and viable cells using fluorescent dyes such as propidium iodide (PI), SYTO 9, and SYTOX Green [175,176] or, alternatively, staining with fluorogenic ester dyes based on the enzymes of living cells; however, these methods are limited in assessing microbial cells [177]. 

The cellular ultrastructure of cyanobacteria undergoes significant changes under RCD, and due to the bacterial size, scanning electron microscopy (SEM) and transmission electron microscopy (TEM) are the major technologies of choice. A number of studies describing the detailed ultrastructure changes during cell death in cyanobacteria are available [37,40,50,72,73]. Distorted cell walls, surface deformation, and enlarged disintegrated thylakoid membranes were observed in the cells. Moreover, cells undergoing RCD appeared to be smaller in size in comparison with controls [36,37,40,50,72,73].

The discovery of RCD-like events in prokaryotes reveals the possibility that widely divergent organisms possess conserved and previously unrecognized RCD pathways [44]. Caspases (cysteine-dependent aspartyl-specific proteases) are a superfamily of conserved, cysteine-dependent, aspartate-directed effector proteases that play an essential role in regulating PCD in metazoans [178,179,180]. Cyanobacteria lack true caspases, and therefore the practice of studying their caspase-like activities using fluorogenic caspase substrates for metazoans is questionable (rev. [66]). 

Most published caspase activity assays in cyanobacteria rely on commercial fluorogenic substrates that produce fluorescence signals upon proteolytic cleavage, indicating the presence of an active caspase [173]. Fluorescence of subsequently cleaved substrate in cyanobacteria in response to H_2_O_2_, salinity changes, and allelopathic stimuli was quantified using a microplate reader or conventional flow cytometer [40,45,50,54,55,73], and the activity of cyanobacterial caspase-like enzymes in some cases was reported to be inhibited by pan-caspase inhibitors (such as zVAD-fmk) [62]. However, the caspase-like homologs in cyanobacteria are more likely to have different specificity for the cleavage site (Arg or Lys) than mammalian caspase homologs and, therefore, would require different specific substrates and inhibitors [132]. The pan-caspase inhibitors (including the most commonly used zVAD-fmk) also inhibit serine non-caspase proteases and *N*-glycanases, inducing autophagy [181,182,183]. 

Recently, the first attempt to compare meta-caspase activity using specific substrates for cyanobacterial caspase-like enzymes and standard substrates for mammalian caspase evaluation was made [184]. The design of specific substrates for caspase-like enzymes in cyanobacteria, using non-specific substrates (such as fluorescein casein), and monitoring overall proteolytic activities may be required [66]. The interpretation of experiments with commercially available mammalian caspase kits should be made cautiously in the absence of complementary assays.

Targeting intracellular components for degradation is a crucial intracellular process that can counteract the effects of external stress such as invading pathogens [185]. The bioinformatic approach helped to explore the distribution and conservation of autophagy-related genes in yeast, photosynthetic eukaryotes, and possibly of their remote homologs in prokaryotes [186,187,188]. In unicellular cyanobacteria regulated degradation of thylakoids may be part of cell survival and not a cell death program due to the need to modulate the photosynthetic apparatus in response to starvation. The classical TEM approach was important in the initial detailed characterization of RCD and RCS mechanisms in cyanobacteria but had limited capabilities to differentiate between these two genetically regulated mechanisms. Further progress in our understanding of RCD would require the implementation of a statistically sound multi-omics analysis of thousands of single cells in combination with cell sorting and approaches allowing the visualization of phenotypic heterogeneity at a single cell level [189,190,191,192]. Single-cell genomics and single-cell transcriptomics target individual pre-selected cells. However, only genes that are expressed above certain thresholds can be sampled. In the future, we expect these thresholds to be significantly lowered, allowing us to go beyond taxonomic diversity research toward quantitative analysis of gene-regulated processes in the unicellular cyanobacteria, cell cycles, physiology, and ecological interactions. Valuable additional information would come from the imaging of cells before transcriptome profiling or whole-genome sequencing, allowing the association of results with specific cell images unambiguously. This approach was applied for single-cell sequencing of marine protists [193,194]. The collaborative efforts would require facilitating steps for the development of new cyanobacteria model systems beyond the existing few. The genetic manipulation systems similar to those developed by the Environmental Model Systems (EMC) initiative [195], which will enable deeper insights into the cell biology of cyanobacteria and the understanding of genetically regulated mechanisms such as RCD and RCS. The major methodological challenge is the current absence of a link between high-throughput genomic and morphological data. A comparison of parallel approaches demonstrates a low degree of overlap between these two approaches [196]. We need technologies that allow image-based cell sorting with a sequential application of genomic methods [197,198]. Cell sorting technologies require classifying cells in real-time [199], and complex phenotypes may require machine learning algorithms for analysis [200].

When we had already submitted our manuscript, we came upon the interesting recent publication of Li and co-authors (2022) proposing to subdivide RCD cell types in cyanobacteria based on the involvement of caspase homologs [201]. In our opinion, the proteolytic system in the unicellular cyanobacteria involved in the highly specific proteolysis and a multi-step repair of the thylakoid complexes with de novo synthesized copies may go far beyond caspase family homologs [202,203].

## 4. Conclusions

RCD in cyanobacteria contributes to increased population survival and is an essential part of the ecological process. The multicellularity for many cyanobacterial clades suggests cell-death-related mechanisms of differentiation and protection against infection from viruses, fungi, and other pathogens. In published research, cyanobacteria species used in RCD studies are mainly represented by multicellular filamentous organisms or colonial and unicellular species having reversed from multicellularity in their evolutionary history. On the other side, there are research studies of regulated cellular survival programs in cyanobacteria, mainly using unicellular species (*Synechococcus* and *Synechocystis* spp.). The key question to be addressed is—are the data obtained with comparative genomics and supporting execution of regulated cell death in multicellular cyanobacteria applicable to unicellular cyanobacteria? We suggest that the enzymes in mechanisms in unicellular cyanobacteria are very different from those in other cyanobacteria groups. In fact, the execution of regulated cellular survival and not a regulated cellular death can be a desired outcome for these bacteria. 

The existing techniques are borrowed from vertebrate RCD research, particularly from mammalian cells equipped with mitochondria—central organelles involved in their process of cell death—and are limited in their capabilities. Further RCD research in cyanobacteria requires the development of proper methods for assessment and wider use of molecular genetics and single-cell analysis in combination with cell sorting based on advanced imaging. This may lead to the discovery of novel RCD molecular pathways and regulatory and inhibitory molecules and contribute to understanding and managing bloom-forming cyanobacteria. 

## Figures and Tables

**Table 1:** Characterization of RCD in cyanobacteria.

Cyanobacterial Strain	Cell Death Inducer	Cell Death Verification							Reference
		Cell Viability	Cell Membrane Rearrangement	Morphological Changes	Caspase-Like Activity	DNA Fragmentation	Photosynthetic ACTIVITY	ROS Production	
*Anabaena flos-aquae*	High irradiance	BacLight viability kit	nd	nd	nd	nd	^14^C labelling	nd	[28]
Field samples *(Synechococcus)*	nd	SYTOX green	nd	nd	nd	nd	nd	nd	[15]
*Anabaena strains (Anabaena* sp.7120, *A. cylindrica, A. siamensis, A. flos-aquae)*	Salt stress	Hoechst 33342, DAPI	nd	TEM	nd	TUNEL, gel electrophoresis	nd	nd	[29]
*Trichodesmium* IMS101*, Trichodesmium erythraeum*	P and Fe starvation, high irradiance, oxidative stress	nd	nd	TEM	Caspase assay	TUNEL	nd	nd	[16]
Lake phytoplankton communities	nd	Enzymatic cell digestion, BacLight viability kit	nd	nd	nd	nd	nd	nd	[30]
*Microcystis aeruginosa*	High salinity, UV irradiance, paraquat herbicide, sonic injury, H_2_O_2_	nd	nd	nd	Caspase-3 assay	nd	nd	DCFH-DA	[31]
*Trichodesmium* IMS101*, Trichodesmium erythraeum*	P and Fe starvation, high irradiance, oxidative stress	nd	nd	nd	Caspase assay	TUNEL	FRR fluorometry	nd	[17]
*Microcystis flos-aquae, Anabaena flos-aquae*	nd	Evans blue, Hoechst 33342	nd	nd	nd	TUNEL	nd	nd	[32]
*Anabaena variabilis* PCC 7937	Irradiance	nd	nd	nd	nd	nd	nd	DCFH-DA	[33]
*Microcystis aeruginosa*	UV-C irradiance	SYBR green, PI	nd	nd	nd	nd	nd	nd	[34]
*Microcystis aeruginosa*	High temperature, darkness, H_2_O_2_	SYTOX green	nd	nd	Caspase-3 assay	TUNEL	FIRe fluorometry	DHR	[35]
*Microcystis aeruginosa* PCC 7005	ß-cyclocitral	SYTOX green, FDA	nd	SEM	nd	nd	nd	nd	[36]
*Microcystis aeruginosa* PCC7806	*Bacillus mycoides*	nd	nd	SEM, TEM	nd	nd	nd	nd	[37]
*Microcystis aeruginosa* FACHB-905	H_2_O_2_	MTT, Hoechst 33342	nd	TEM	Caspase-3 assay	TUNEL	PAM fluorometry	nd	[18]
*Microcystis aeruginosa* UTEX B 2667	H_2_O_2_	FDA, SYTOX green	nd	nd	nd	nd	High time-resolution fluorometry	nd	[38]
*Trichodesmium* IMS101	nd	nd	nd	nd	Caspase assay	nd	nd	nd	[39]
*Cyanobacterium living endophytically in the fern Azolla microphylla*	*Azolla microphylla* subjected to darkness, nutrient starvation, -radiation	nd	Annexin V	TEM	nd	TUNEL	nd	nd	[11]
*Microcystis aeruginosa* FACHB-905	*Myriophyllum spicatum*	Cell counting using flow cytometry	nd	TEM	Caspase-3 assay	Gel electrophoresis	nd	DCFH-DA	[40]
*Anabaena 7120*	High temperature	DAPI	nd	nd	nd	nd	nd	nd	[41]
*Microcystis aeruginosa*	Glyphosate	Apoptosis assay kit	nd	nd	nd	nd	nd	nd	[42]
*Aphanizomenon* sp.	nd	SB (IFC)	Annexin V (IFC)	nd	nd	nd	nd	H_2_DCFDA	[43]
*Microcystis aeruginosa* TAIHU98	Prodigiosin from *Hahella* sp. KA22	DAPI	PI, Annexin V	SEM, TEM	nd	Gel electrophoresis	PAM fluorometry	DCFH-DA	[44]
*Microcystis aeruginosa* DIANCHI905	Pyrogallic acid from *Myriophyllum spicatum*	nd	PI, Annexin V	TEM	Caspase-3 assay	Gel electrophoresis	nd	DCFH-DA	[45]
*Synechocystis*	High temperature	SYTOX green	nd	nd	nd	nd	nd	nd	[46]
*Anabaena fertilissima*	High salinity	DAPI	Annexin V	nd	nd	Gel electrophoresis	nd	DCFH-DA	[47]
*Microcystis aeruginosa* FACHB-905, FACHB-915	Low temperature, darkness	MTT	nd	nd	Caspase-3 assay	TUNEL	PAM fluorometry	nd	[48]
*Microcystis aeruginosa* HYK0906-A2	Naphthoquinone derivative NQ 2-0	nd	nd	TEM	nd	nd	PAM fluorometry	DCFH-DA	[49]
*Microcystis aeruginosa* FACHB-905	H_2_O_2_	SYBR green, PI	nd	SEM	Caspase-3 assay	nd	nd	H_2_DCFDA	[50]
Field samples *(Trichodesmium)*	nd	nd	nd	nd	Caspase assay	nd	nd	nd	[51]
*Microcystis aeruginosa* MGK	H_2_O_2_	nd	nd	nd	nd	nd	PAM fluorometry	nd	[52]
Water samples (*Microcystis aeruginosa*)	nd	SYTOX green	Annexin V	nd	nd	nd	nd	nd	[53]
*Microcystis aeruginosa* LB2385	High salinity	SYTOX green	nd	nd	Caspase-3 assay	nd	PAM fluorometry	DCFH-DA	[54]
*Trichodesmium erythraeum IMS101,* field samples	Fe starvation, high irradiance	nd	nd	nd	Caspase assay	nd	nd	nd	[55]
*Microcystis viridis* FACHB-979	Glyphosate	Apoptosis assay kit	nd	nd	nd	nd	nd	nd	[56]
*Microcystis aeruginosa* FACHB-905	H_2_O_2_, UV-C irradiance	SYBR green, PI, DiOC6	nd	nd	nd	nd	nd	nd	[57]
*Halothece* sp. PCC 7418*, Fischerella muscicola* sp. PCC 73103	Nutrient (P, Fe) starvation	nd	Annexin V	TEM	nd	nd	nd	nd	[58]
*Microcystis aeruginosa* SAG14.85	*Daphnia* grazers	nd	nd	nd	Caspase assay	nd	nd	DCFH-DA	[59]
*Microcystis aeruginosa* FACHB-905	Eugenol	nd	nd	TEM	nd	nd	Fluorometry	nd	[60]
*Microcystis aeruginosa* FACHB-912	High irradiance, high temperature	nd	nd	nd	nd	nd	nd	DCFH-DA (IFC)	[61]
*Microcystis aeruginosa* FACHB-905	H_2_O_2_	SYBR green, PI	nd	SEM, TEM	Caspase-3 assay	TUNEL	PAM fluorometry	nd	[62]
*Synechocystis* sp. PCC 6803	Heat stress	SYTOX green, FDA	nd	TEM	Caspase assay	nd	nd	H_2_DCFDA	[63]
*Microcystis aeruginosa* CAAT2005-3	H_2_O_2_	SYTOX green	nd	TEM	nd	nd	nd	DCFH-DA	[64]

nd-not determined.
